# The Material Value of Æsthetics

**Published:** 1918-03-23

**Authors:** Hubert J. Norman


### THE MATERIAL VALUE OF ÆSTHETICS.

By HUBERT J. NORMAN, M.B., CH.B.(ED.).

It is a common assumption of those who decry the bogey which they denominate "materialism" that, just as there is no place in its scheme of things for "invisible kings" and suchlike mystical abstractions, so there cannot be any value attached by it to those things which may be included in the category of æsthetics—art, literature, music, etc. Since it is no longer possible actually to persecute those who hold unorthodox opinions—the power, not the will, to do so being in default—it is found advisable to set up a man of straw and then to pound him with hard words instead of with stones. The fallacy is, however, just as great as is that which is based upon the belief that, once the hope of reward or the fear of punishment in the hereafter is shown to be without reasonable support, mankind will tend to anarchy or to entirely self-centred hedonism. Experience shows that those who believe that their chief duty lies in observance of all the implications of the Golden Rule, and that social errors bring their punishment in this life, are not one whit worse citizens than those whose other-worldliness leads them at times to neglect as of minor importance their responsibilities towards their fellow-worms!

Looked at from the broad biological point of view, the human body is not something utterly separate, not is there any personality entirely free and self-sufficient. In the slow process of evolution man has been moulded and fashioned by stimuli reaching him from his environment, beginning, as the study of development of the individual makes clear, as a simple monocellular structure and gradually passing through stages of increasing complexity. During the life of the race an incalculable number of stimuli of varying degrees of intensity have been necessary to achieve the result seen as civilised man. These stimuli have served also to give rise to those apparently simple structures which, given suitable environment, have within them the potentiality to develop into new individuals.

It is well to note that those microscopic bodies, the ovum and the spermatozoon, from the coalescence of which the new individual has its inception, have been spoken of as "apparently simple." Analogy, however, will help us to realise that size is no criterion of complexity, and, granted the atomic theory of matter, even in a microscopic speck there may be a whole microcosm of atoms and energies. This is exemplified again in those minute micro-organisms which live out their career in so small a space that only a very high power of the microscope can reveal them to us. Taking the individual as arising in the coalescence already mentioned, it is seen that, from the very first, stimuli are acting upon it from the maternal blood-supply and the substances circulating therein, and in this way, through maternal indiscretions or accidents, noxious stimuli may serve to prejudice the growing organism even during pre-natal life. It is not necessary to dwell at length here on ante-natal conditions, but it is essential to mention them because of their undoubted influence, and also because they are too often left out of account in considering the history of the individual.*

From birth onwards the body is subjected to constantly recurring stimuli of a more complicated nature than those which act upon it during intra-uterine life. The special senses, and the areas of the brain whither the stimuli which impinge upon them are conveyed, gradually become educated. This education is obviously dependent upon the two chief factors—the environmental conditions and the quality of the cerebral tissue. But the development of the brain is influenced also by the more circumscribed environment which is contained in the body itself. Thus from the internal organs and from the substances secreted by various glands it receives constant stimuli; and certain of these secretions are of such importance—for instance, that from the thyroid gland—that without them mental development cannot take place. Indeed, for those who still hold the belief of a "mind" entirely separate from bodily structure, but at the same time having its seat in some part of the body, it would not be amiss to modify the Cartesian doctrine and place the soul in the thyroid instead of in the pineal gland.

In process of time the nervous system becomes adapted to the influence of stimuli which in earlier stages would have failed to influence it. As water flowing over the land takes the path of least resistance and gradually makes for itself channels through which it can move with more ease and at greater speed, so in the brain canalisation takes place, and stimuli pass more readily along these beaten tracks. Again, the passage of the water and the formation of channels depend upon the conformation and the structure of the land; and in the brain the result depends not only on the stimuli applied, but on the quality of the nervous system which is stimulated.

Biologically the body is hedonistic. It is the pleasurable stimuli—or rather, those which give rise to feelings of pleasure or gratification—which must preponderate over those which cause harm or which produce from the psychological point of view pain or displeasure. It is the former which tend to build up or to integrate, while the latter bring about destruction or disintegration. Even with the former it is, however, a question of degree; and only pleasurable stimuli of a certain degree of intensity are beneficent in their effects. Beyond that point they become dangerous and productive of disorder. Herein lies the difficulty of those who wish to define categorically such terms as pleasure or pain, good or evil.

In the last resort the stimuli which act upon the body by means of its nervous end-organs can be

* Especially at the present time in regard to the induction of "twilight sleep" by means of potent drugs, which certainly appear to have no good influence on the offspring.

**MATERIAL VALUE OF ÆSTHETICS—*(continued)*.**

resolved into comparatively simple components. What is a Beethoven symphony but a series of sound-waves of orderly arrangement impinging upon the tympanum and being conveyed thence through the other channels of the ear to the brain? Or a Turner landscape but a series of light waves impinging upon the retina and passing thence centripetally? The resulting pleasurable effect is only brought about in the individual who has been trained—that is, whose nerve-cells have been gradually educated by the previous passage of an enormous number of stimuli of a similar character. Where such a condition has been brought about the incoming fresh stimulus harmonises with those which have gone before, and a pleasurable feeling results. Discords or crude colourings we speak of as " setting our nerves on edge," and this popular manner of expressing the effect really describes the physiological result. But we cannot draw up any definite rules as to æsthetic value from the common psychological point of view, whether the matter under consideration is music, literature, or art. Different individuals have their own criteria. Nor is it just to restrict our view in æsthetics to the sensations derived from certain nerve-terminals, such as the eye and the ear. Other receptors, such as those in the mouth and the nose, the general sensibility of the skin, the internal stimuli coming from within the body, are not less worthy of notice. Only in a purely artificial classification is it possible to put these in a separate category. Indeed, where some of the special senses are unable to fulfil their functions—as, for example, the eye and the ear—the general skin sensibility may be educated to respond in a remarkable way to stimuli which otherwise would not be appreciated.*

From all these sources a variety of stimuli are conveyed to the brain, and have their effect therein upon the nerve-cells and upon the vascular supply. For convenience of description certain of them have been divided from the rest, and have been denominated psychological in contradistinction to the others which are spoken of as physiological. Of the former, some which stimulate certain areas in a manner which, on the subjective side, gives rise to pleasurable feelings are described as æsthetic by those individuals in whose consciousness they emerge as pleasant sensations. But, as has been said, it is not possible thus to separate biologically the one set of stimuli from the other. It is simply a question of whether they act favourably upon the organisation or are inimical to it. If the former, then the stimuli tend to maintain the organism in a healthy state, or at least not to bring about rapid wastage of nerve tissue; if the latter, they will speedily, if unintermitted, bring about disintegration and death.

* When we remember that the eye and the ear are, as embryological research has shown, only specialised areas of the ectoderm or original outer layer, it becomes more easy to understand how this is possible.

